# Improved Hydrophobicity and Dimensional Stability of Wood Treated with Paraffin/Acrylate Compound Emulsion through Response Surface Methodology Optimization

**DOI:** 10.3390/polym12010086

**Published:** 2020-01-03

**Authors:** Jun Jiang, Yupeng Chen, Jinzhen Cao, Changtong Mei

**Affiliations:** 1 College of Materials Science and Engineering, Nanjing Forestry University, Nanjing 210037, China; jiangjun@njfu.edu.cn; 2 MOE Key Laboratory of Wooden Material Science and Application, Beijing Forestry University, Beijing 100083, China; chenyupeng1115@163.com

**Keywords:** paraffin, acrylate, wood treatment, hydrophobicity, dimensional stability, response surface methodology

## Abstract

Wood treatment was conducted by paraffin/acrylate compound emulsion. Response surface methodology (RSM) was applied for modeling and to determine the relationship between hydrophobicity and influencing factors. The results showed that the paraffin emulsion concentration and acrylate emulsion percentage had significant influences on water absorption (WA) and mass percentage gain (MG). The WA decreased obviously with increasing acrylate emulsion percentage. The correlation models for WA and MG showed a good prediction due to the straight-line distribution in the normal probability plot of residuals. The optimal conditions (5.57% paraffin emulsion concentration, 20% acrylate emulsion percentage, and 10 min treatment time) provided by RSM were acceptable for predicting the MG and WA. Compared to untreated (66°) and paraffin emulsion treated wood (94°), the wood treated by compound emulsion showed the highest water contact angle (133°) and better dimensional stability. This could be ascribed to a synergistic effect (bulking effect and filling effect) provided by paraffin and acrylate, which could form a completely hydrophobic film in wood.

## 1. Introduction

As a renewable and natural material, wood has been an essential material for human society. However, due to the chemical components of wood, unprotected wood in outdoor applications easily undergoes anisotropic swelling or shrinkage and is susceptible to chemical or biological degradation. The improvement of hydrophobicity and dimensional stability in wood has been a permanent research challenge for the sake of a better utilization of this environmentally friendly material [1,2]. The impregnation of wood with paraffin emulsion is a popular approach to reduce its water uptake, and a higher paraffin uptake improves the efficiency of this treatment. Furthermore, both the photostability and decay resistance of wood could be improved by this method [3,4]. In our previous studies [5,6], paraffin Pickering emulsion was successfully prepared to treat solid wood and wood flours for composites preparation, through which the hydrophobicity, thermal stability and surface hardness in wood and composites improved obviously. Paraffin emulsion is a dispersion in which the hydrophobic paraffin is homogeneously dispersed in water in the form of small particles with the help of certain surfactants. It has been widely used in wood industries to improve water repellency in wood. For instance, it is reported that a 2.5 wt % paraffin emulsion significantly decreases the water absorption in wood [7]. Moreover, some studies stated that wood impregnation with paraffin emulsion could remarkably improve its mechanical properties [8]. However, due to its chemical inertness, paraffin cannot form a fully water resistant hydrophobic film on a wood surface via chemical reactions to protect wood components from water attack. The physical adhesion or filling effect in wood pores is determined to improve the hydrophobicity of paraffin treated wood [5,9].

Compared to paraffin emulsion, acrylate emulsion is produced through polymerization of monomers, such as esters of acrylic or methacrylic acid. It has a broad application in adhesives, paints, varnishes, impregnated papers, and wood industries [10]. Additionally, wood impregnation with multifunctional monomers could form chemical bondings between modifiers and the wood matrix to reduce the number of hydroxyl groups within cell walls, resulting in improved hydrophobicity, decay resistance and mechanical properties [11]. A radical polymerization process leads to the formation of various types of polymers that could protect the wood matrix against water. It is reported that introducing methyl methacrylate and styrene into the wood structure through a vacuum/pressure technique and a subsequent catalyst thermal process would lead to improvements in dimensional stability, thermal stability and decay resistance [12]. Long et al. [13] prepared acrylate emulsion with nano-alumina slurry for wood coating. When a 1.5 wt % nano-alumina slurry was mixed with acrylate emulsion, the wood surface showed improvements in abrasion resistance and hardness. Hoque et al. [14] and Islam et al. [15] prepared wood/poly (methyl methacrylate) composites by impregnating wood with methyl methacrylate monomers, through which improvements in water repellency, dimensional stability, and resistance against biodegradation were obtained. On the other hand, acrylate emulsions, due to their oil-in-water (O/W) emulsion type, and other water-based modifiers are miscible, and the resulting compound modifiers could bring some synergistic improvements in anti-weathering, hydrophobicity, and decay resistance of wood [16].

Therefore, a combined treatment with paraffin emulsion and acrylate emulsion seems to be a promising method to synergistically improve wood hydrophobicity and dimensional stability. Owing to the presence of acrylate, the film formation of paraffin might be improved. In this study, paraffin emulsion and acrylate emulsion were physically mixed as a compound emulsion for wood treatment. The acrylate emulsion was used to modify paraffin emulsion by physical blending. The objective of this study is to investigate the effects of paraffin emulsion concentration, acrylate emulsion percentage, and treatment time on the hydrophobicity and mass percentage gain (MG) of treated wood via response surface methodology (RSM). RSM is known as an effective mathematical and statistical tool to evaluate the effects of independent variables and their interactions on responses. In addition, a comparative study was conducted to evaluate the optimal condition obtained by RSM. The water absorption (WA) and mass percentage gain in compound emulsion treated wood were calculated to evaluate the validation of the prediction. The water contact angle (WCA), dimensional stability and microstructure of untreated wood and wood treated by different emulsion systems (paraffin emulsion and compound emulsion) were also investigated to clarify the effect of acrylate emulsion addition on the hydrophobicity of paraffin emulsion treated wood.

## 2. Materials and Methods

### 2.1. Materials

High purity liquid paraffin (≥99%), Tween 80 (HLB = 15) and Span 80 (HLB = 4.3) as surfactants were purchased from Beijing Chemical Ltd., Beijing, China. Acrylate emulsion BA-201, 10% was provided by Beijing Donglian Chemical Ltd., Beijing, China. Southern pine (*Pinus* spp.) sapwood with an air-dried density of 0.43 g cm^−3^ and an average growth ring width of 0.6 cm was collected from the northeast of China.

### 2.2. Preparation of O/W Paraffin Emulsion

According to our previous study [5], Tween 80 (1.3 wt % based on water mass) and Span 80 (1.5 wt % based on water mass) were added to deionized water and gently stirred at a speed of 500 rpm for 30 s. Then, the oil phase (1:5 by vol.) was added into the mixture and pre-emulsified at 5000 rpm for 2 min. Afterwards, the pre-emulsion (about 800 g) was further emulsified at 45 MPa for 5 min in a high-pressure homogenizer (APV-2000, APV Manufacturing Poland Sp.z.o.o., Bydgoszcz, Poland).

### 2.3. Preparation of Paraffin/Acrylate Compound Emulsion

Prior to the addition of acrylate emulsion, the paraffin emulsion was diluted to prepare 2 wt %, 5 wt % and 8 wt % emulsions. Then, 10 wt % and 20 wt % acrylate emulsions were added (based on the weight of paraffin emulsion) and gently stirred at 1000 rpm to obtain a homogeneous compound emulsion. Different combinations of compound emulsions are listed in Table 1. The droplet size of the emulsion was determined by a laser particle size analyzer (Delsa Nano C, Beckman Coulter, Atlanta, GA, USA). Three light scattering measurements were conducted for each sample, and the average values are reported in Table 1.

### 2.4. Wood Treatment and Characterization

The wood samples were treated with the paraffin emulsion and compound emulsion through a vacuum/pressure process. Samples with dimensions of 20 (L) × 20 (R) × 20 (T) mm^3^ were first evacuated at −0.09 MPa for 30 min and then pressurized at 0.6 MPa for 45 min. After impregnation, the excessive emulsion was removed by a clean towel and then samples were oven-dried at 60 °C for 24 h. Afterward, the samples were cured at 103 °C to reach a constant weight. Mass percentage gain (MG) was calculated using the following equation, with six replicates for the mean value:

(1) $$\mathrm{MG}\;\left(\%\right)=\frac{m_{0}^{\prime }-m_{0}}{m_{0}}\times 100\%$$

where $m_{0}^{\prime }$ is the oven-dried weight after treatment, and *m*_0_ is the oven-dried weight before treatment. Six replicates were performed for each sample, and the mean values were calculated.

For water absorption (WA) tests, the dimensions of the samples were 20 (L) × 20 (R) × 20 (T) mm^3^. The immersion time in deionized water was 48 h, and the averages of six replicates were calculated. The following equations were used to calculate the water absorption:

(2) $$\mathrm{WA}\;\left(\%\right)\;=\;\frac{m_{1}-m_{0}}{m_{0}}\;\times \;100\%\;\;\;\;\left(\mathrm{untreated}\;\mathrm{wood}\right)$$

(3) $$\mathrm{or}\;\;\mathrm{WA}\;\left(\%\right)\;=\;\frac{m_{1}-m_{0}^{\prime }}{m_{0}^{\prime }}\;\times \;100\%\;\;\;\left(\mathrm{treated}\;\mathrm{wood}\right)$$

where *m*_1_ refers to the oven-dried weight after immersion.

After immersion tests, the dimensional stability of untreated and treated wood samples was determined according to the Chinese standard GB/T 1928–2009. The dimension changes in tangential direction, radial direction and longitudinal direction of samples were measured. The tangential swelling ratio (TSR), radial swelling ratio (RSR), and longitudinal swelling ratio (LSR) were calculated using the following equations, with five replicates for each sample:

(4) $$\mathrm{TSR}\;=\;\frac{T_{1}-T_{0}}{T_{0}}\;\times \;100\%$$

(5) $$\mathrm{RSR}\;=\;\frac{R_{1}-R_{0}}{R_{0}}\times 100\%$$

(6) $$\mathrm{LSR}\;=\;\frac{L_{1}\;-L_{0}}{L_{0}}\times 100\%$$

where *T*_1_, *R*_1_, and *L*_1_ are the dimensions in different directions after immersion; and *T*_0_, *R*_0_, and *L*_0_ are the dimensions of oven-dried samples in different directions before immersion.

For water contact angle (WCA) measurements, the sessile drop method was applied (OCA20, DataPhysics Instruments GmbH, Filderstadt, Germany). Droplets of deionized water were placed on planed cross-sections of the sample treated with different emulsions, and the WCAs were observed by a built-in digital camera after 30 s. The average values of the three replicates were reported.

The micromorphology of the samples before and after treatment with different emulsions was observed by scanning electron microscopy (SEM) (S-3400, Hitachi, Tokyo, Japan, 10 kV). Before observation, all samples were dried at 103 °C and the transverse surfaces of samples were coated with a thin layer of Pt-Pb. The distribution of emulsion in the wood was observed.

### 2.5. Response Surface Methodology

A Box–Behnken design (BBD) was applied to investigate the effect of acrylate emulsion on the MG and WA of treated wood. Three factors and their levels were chosen according to the authors’ previous studies [5,17] and are shown in Table 2. In this research, fitted second-order polynomial regression analysis was utilized to obtain the approximation of the response, and the following model was derived:

(7) $$Y=b_{0}+\;\sum_{i=1}^{k}b_{i\;\;}X_{i}+\sum_{i,j}^{k}b_{ij}X_{i}X_{j}+\;\sum_{i=1}^{k}b_{ii}X_{i}^{2}$$

where *b*_0_ is the free term of the regression equation, *b*_1_, *b*_2_, …, *b_k_* and *b*_11_, *b*_22_, …, *b_kk_* coefficients are the linear and the quadratic terms, respectively, and *b*_12_, *b*_13_, …, *b_k−_*_1_ *k* are the interaction terms.

**Scheme 1:**
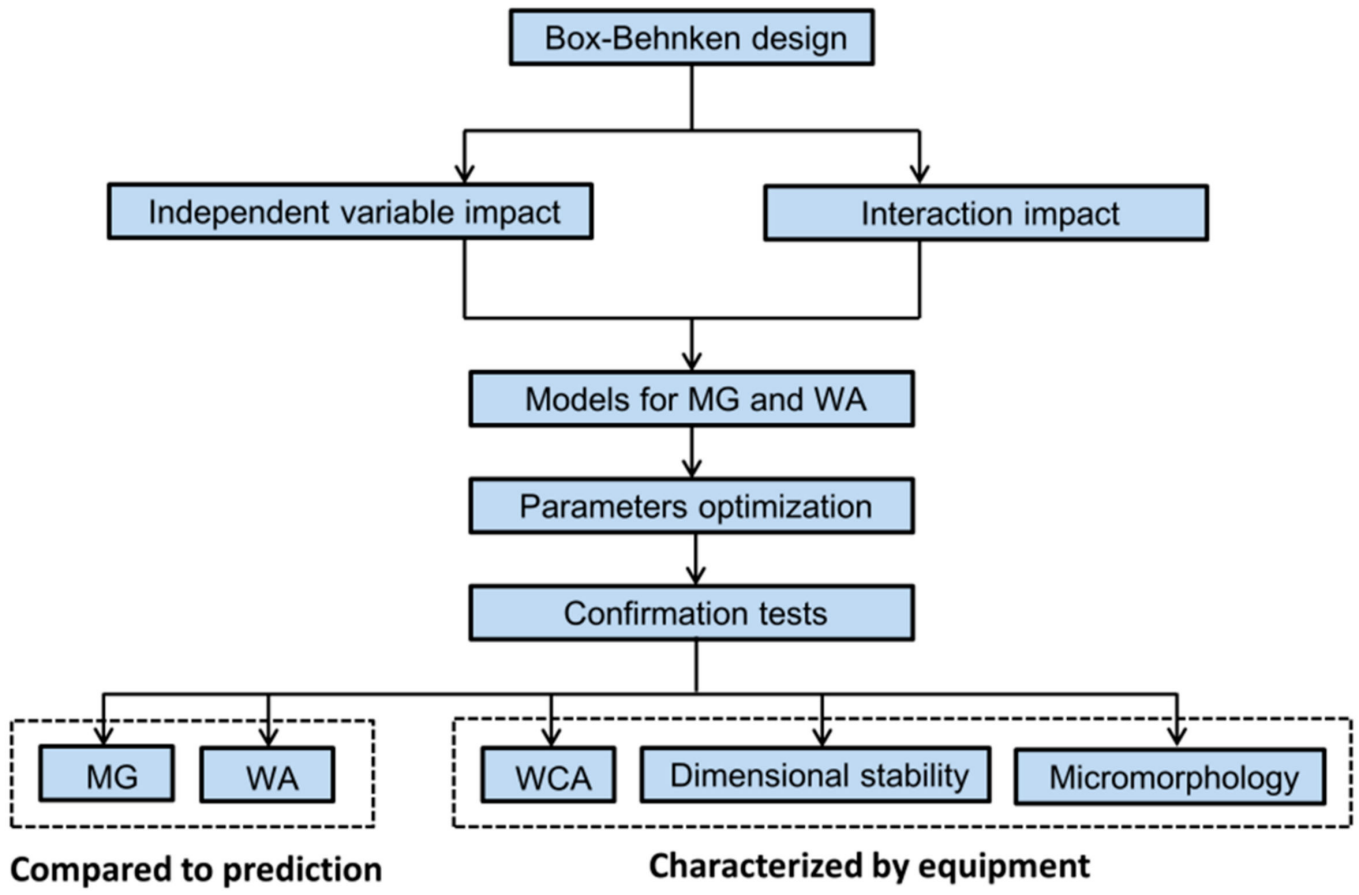
Diagram of the experimental design (MG: mass percentage gain; WA: water absorption; WCA: water contact angle).

In order to conduct RSM analysis, a regression was performed on the collected data, wherein the observed variable (response) was approximated based on a functional relationship between the estimated variables and one or more input variables [18,19,20,21]. The least square technique was used to fit a model equation containing the input variables by minimizing the residual error measured by the sum of square deviations between the actual and predicted response. This involved the calculation of estimations for regression coefficients. The calculated coefficients for the model equation were tested for statistical significance by testing the significance of the regression model, the significance of individual model coefficients and the lack-of-fit. Moreover, the adequacy of the model was investigated by examining the residuals, which are the differences between the observed and predicted values [22]. This was performed by using the normal probability plots of residuals to clarify whether or not the model is adequate to predict the responses. The experimental strategy is shown in Scheme 1.

## 3. Results and Discussion

### 3.1. Analysis of Variance for MG

Table 3 shows the operational conditions and results for MG and WA responses. To avoid order effects, experiments were conducted randomly in five repetitions each and mean values were calculated.

**Figure 1:**
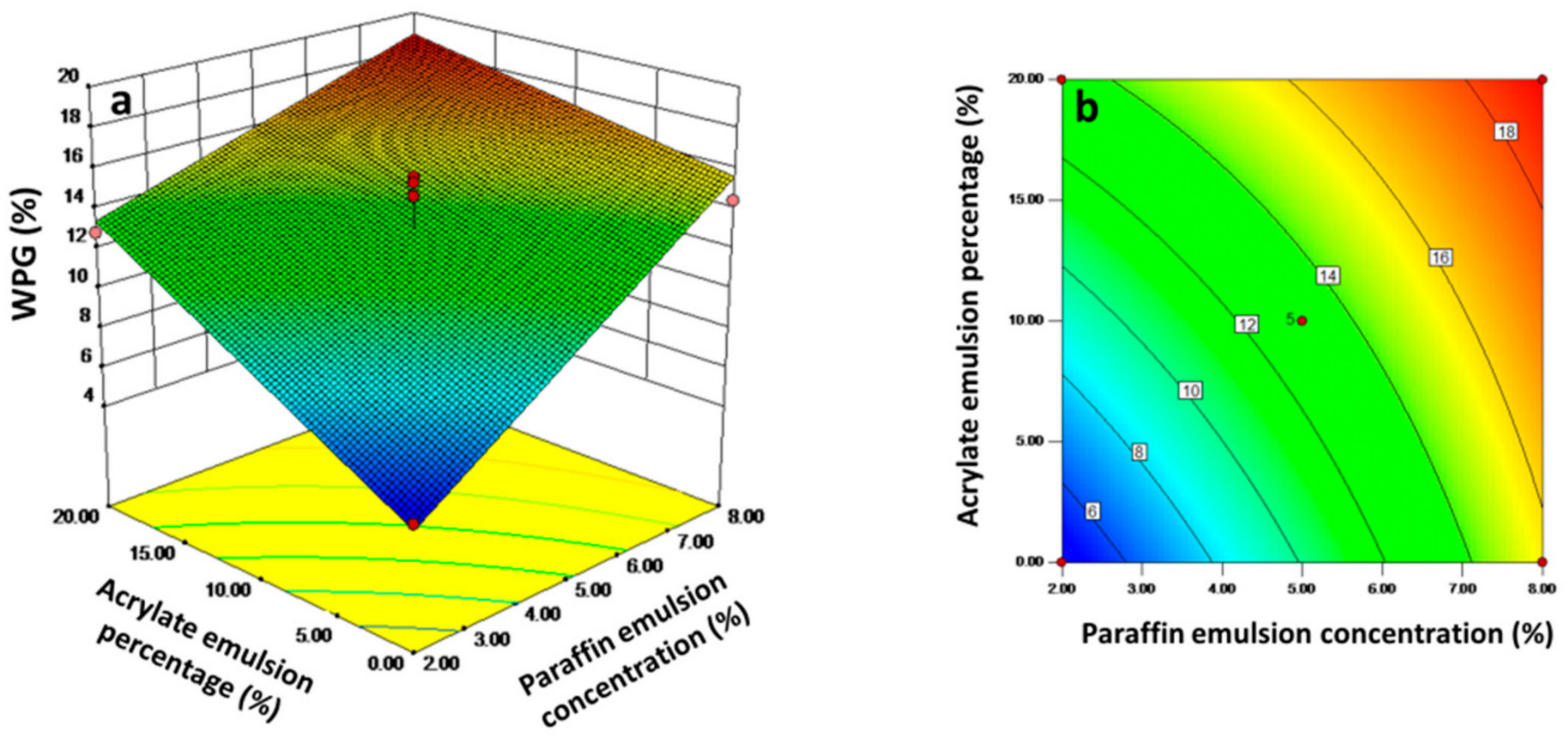
Effects of paraffin emulsion concentration and acrylate emulsion percentage on MG: (**a**) 3D and (**b**) contour surface plots.

Analysis of variance (ANOVA) was carried out to evaluate the effectiveness of a quadratic model for testing the statistical significance of the BBD model. As shown in Table 4, the selected model for MG was highly significant, with a *P*-value < 0.01. As can be seen in Table 4, variables A and B have dominant contributions to MG, among which the paraffin emulsion concentration (A) showed a greater impact on the MG (*P*-value < 0.0001). By contrast, the treatment time (C) showed a minor effect on the MG (*P*-value > 0.1). This could be attributed to the difference between the droplet size of the compound emulsion and the pore size in the wood structure (micropores < 2 nm, mesopores 2–50 nm and macropores > 50 nm). In other words, the emulsion could penetrate into the wood structure much faster when the droplet size was smaller than the pore size and as a result, the MG could be affected by treatment time. However, if the droplet size of the emulsion is bigger than the pore size, treatment time might show a slight impact on MG [23]. The interaction effects of treatment parameters (AB, AC and BC) did not exhibit a considerable effect on MG. Additionally, lack-of-fit showed a negligible effect on MG, indicating that it is not significant compared to the pure error. The effects of paraffin emulsion concentration and acrylate emulsion percentage are illustrated in Figure 1. As shown below, increasing the paraffin emulsion concentration and acrylate emulsion percentage led to a consistent increase in MG. These findings could play an important role in controlling the MG during wood treatment.

### 3.2. Regression Equation for MG

A response surface equation for MG in compound emulsion treated wood was obtained by Design-Expert software. As shown in Table 5, a model was selected automatically to introduce a relationship between MG and treatment parameters, considering the higher values of the coefficient of determination (R^2^). The R^2^ was calculated as 0.8290, indicating that the model fitted for 82.9% of MG. This showed a good fitness with the experimental results and could satisfactorily explain the effects of the variables on responses. As a porous biomaterial, the pore size distribution in wood could affect the penetration and distribution of compound emulsion, which in turn, could influence the R^2^ to some extent [23]. The extracted model cllows:

(8) $$\begin{array}{c} \mathrm{MG}=-0.96745+2.06333A+0.65208B+0.070833C-0.0475\mathrm{AB}\\ -8.333\times 10^{-3}\mathrm{AC}-4.433\times 10^{-3}\mathrm{BC} \end{array}$$

**Figure 2:**
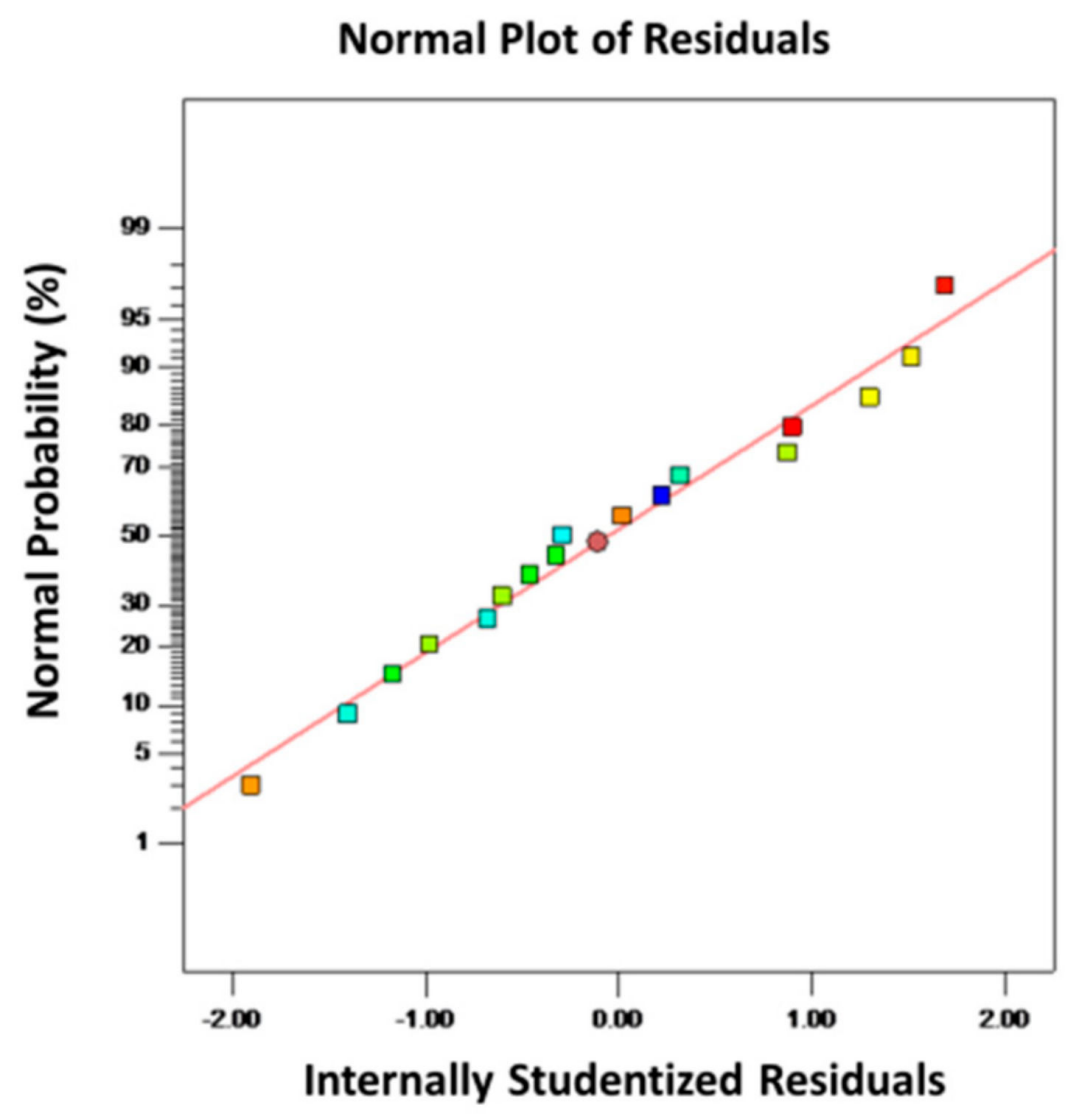
Normal probability plot of residuals for MG.

The normal probability plot of residuals for MG is shown in Figure 2. It reveals that the residuals generally fall on a near straight line, showing that the errors are distributed normally [24,25]. This indicates that the model can effectively predict MG in compound emulsion treated wood.

### 3.3. Analysis of Variance and Model for WA

Analysis of variance (ANOVA) was carried out to evaluate the effectiveness of the model for testing the statistical significance (Table 6). The statistical parameters including *F*-values, coefficient of determination (R^2^), and adjusted R^2^ are summarized in Table 7. The high *F*-values (12.77–40.55) indicated that the model and treatment parameters (variables A and B) have statistically significant influences on WA. Similar conclusions based on *F*-values could be found in the previous study [26]. Considering *P*-value, the paraffin emulsion concentration and acrylate emulsion percentage showed greater impacts on WA compared to treatment time. The interaction effects of treatment parameters (AB, AC and BC) showed minor effects on WA. These findings are in agreement with the analyses for MG in the previous section (Section 3.1). Moreover, the *P*-value for lack-of-fit was not considerable (*P*-value> 0.1), indicating that the distribution of experimental data was a model-independent measure of the pure error. The derived model equation for WA is as follows.

(9) $$\begin{array}{c} \mathrm{WA}=+54.45025+1.46458A-0.53567B+0.012250C-0.23667\mathrm{AB}\\ -8.8333\times 10^{-3}\mathrm{AC}-5.333\times 10^{-4}\mathrm{BC} \end{array}$$

According to Table 7, the R^2^ value for the model (0.8763) showed that the model could well explain the experimental results. Also, the adjusted R^2^ (0.8478) suggested that there was a high degree of correlation between the experimental and predicted values.

**Figure 3:**
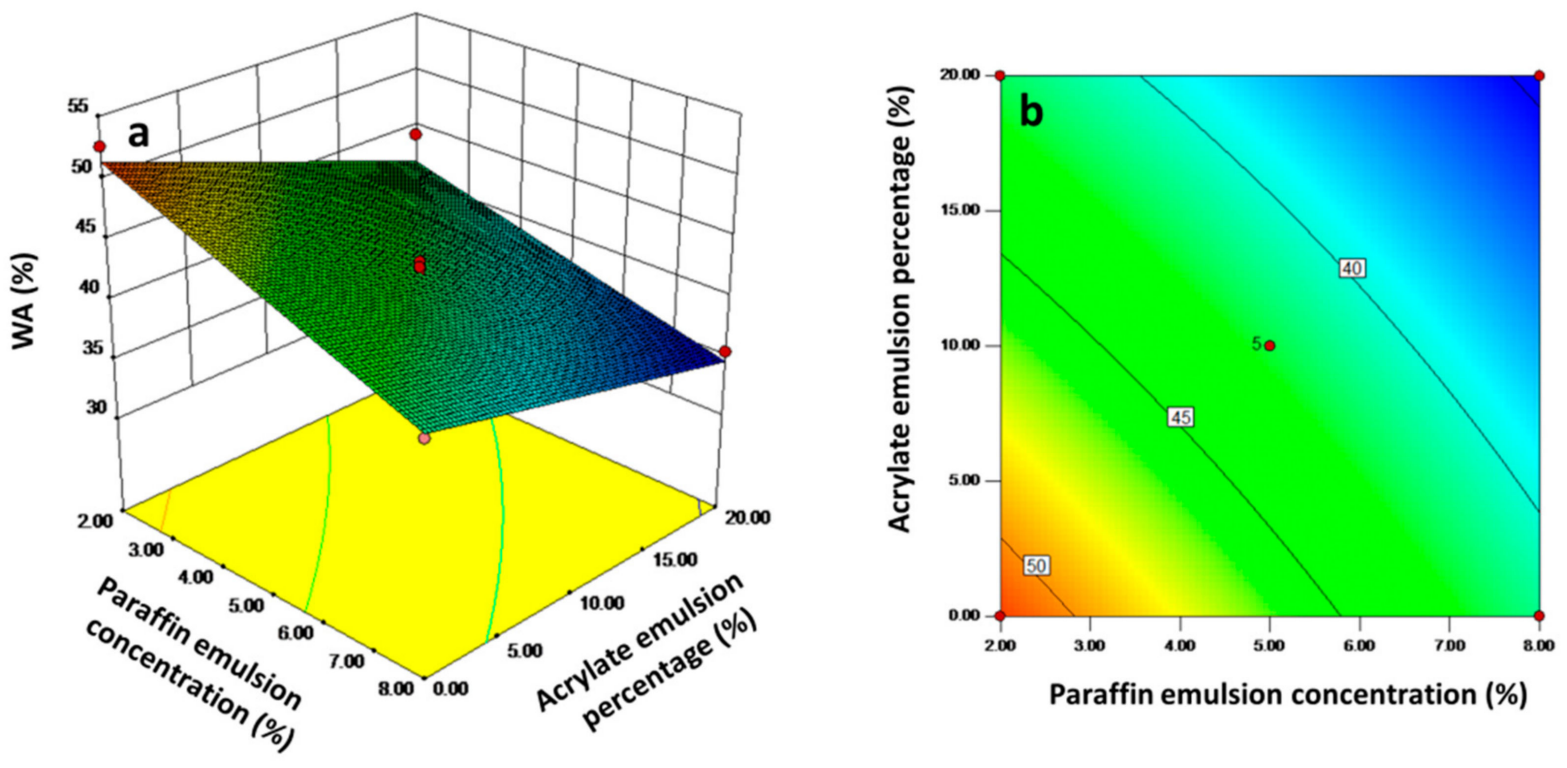
Effects of paraffin emulsion concentration and acrylate emulsion percentage on WA: (**a**) 3D and (**b**) contour surface plots.

Figure 3 shows the surface response of WA as a function of paraffin emulsion concentration and acrylate emulsion percentage. The WA decreased with increasing paraffin emulsion concentration and acrylate emulsion percentage. This could be attributed to the hydrophobicity of paraffin. Furthermore, acrylate emulsion could form a complete polymer film during the drying process (post-treatment after emulsion impregnation) in wood and prevent water penetration [27,28]. Therefore, a synergistically hydrophobic effect provided by paraffin and acrylate emulsion improves the hydrophobicity of treated wood.

**Figure 4:**
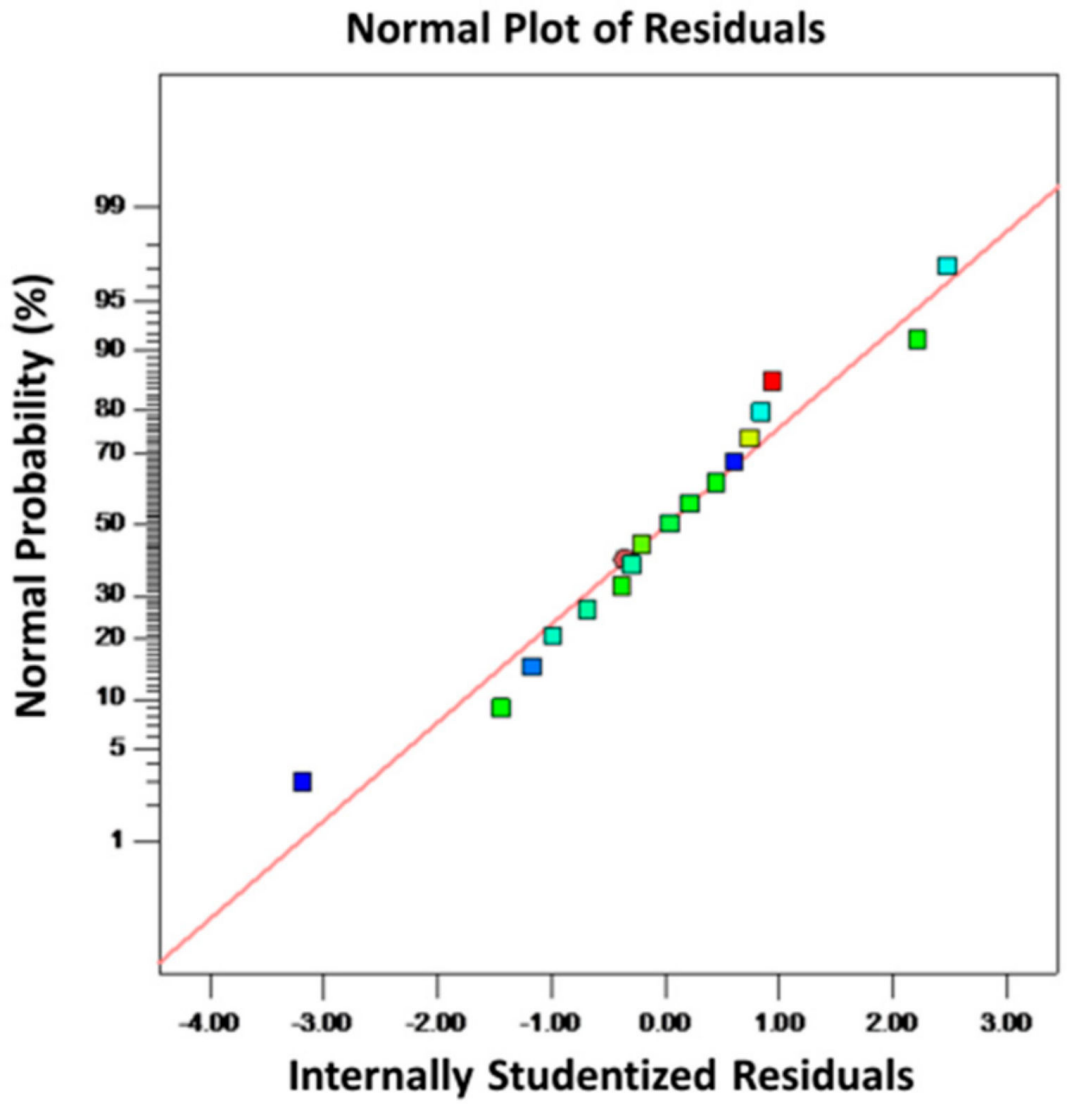
Normal probability plot of residuals for WA.

The normal probability plot of residuals for WA is shown in Figure 4. The residuals generally fall on a near straight line, which means the errors are distributed normally. This suggests that the proposed model is satisfactory. It further indicates that the model can effectively predict the WA in compound emulsion treated wood.

### 3.4. Parameter Optimization and Validation Confirmation Tests

Optimization of the variables was performed to maximize MG and minimize WA, simultaneously. The optimal experimental conditions predicted by RSM are: paraffin emulsion concentration of 5.57%, acrylate emulsion percentage of 20% and treatment time of 10 min. The resulting MG and WA were 17.6% and 37.9%, respectively. Experiments under optimal conditions were carried out to validate the prediction. The experiments were done in three replicates, and the mean predicted and experimental values for the two responses are shown in Table 8.

The experimental data were close to the predicted values, and the error precision was about 10%. This error could be attributed to the hierarchical structure of wood with different sizes of pores, resulting in different distributions of compound emulsion, which could affect the MG and WA. However, it could be said that the optimization was reasonable. This was because all the actual values for the confirmation run were within a 90% prediction interval (about 10% errors), while the R^2^ for the MG and WA models was 82% and 87%, respectively.

### 3.5. Water Contact Angle and Dimensional Stability

**Figure 5:**
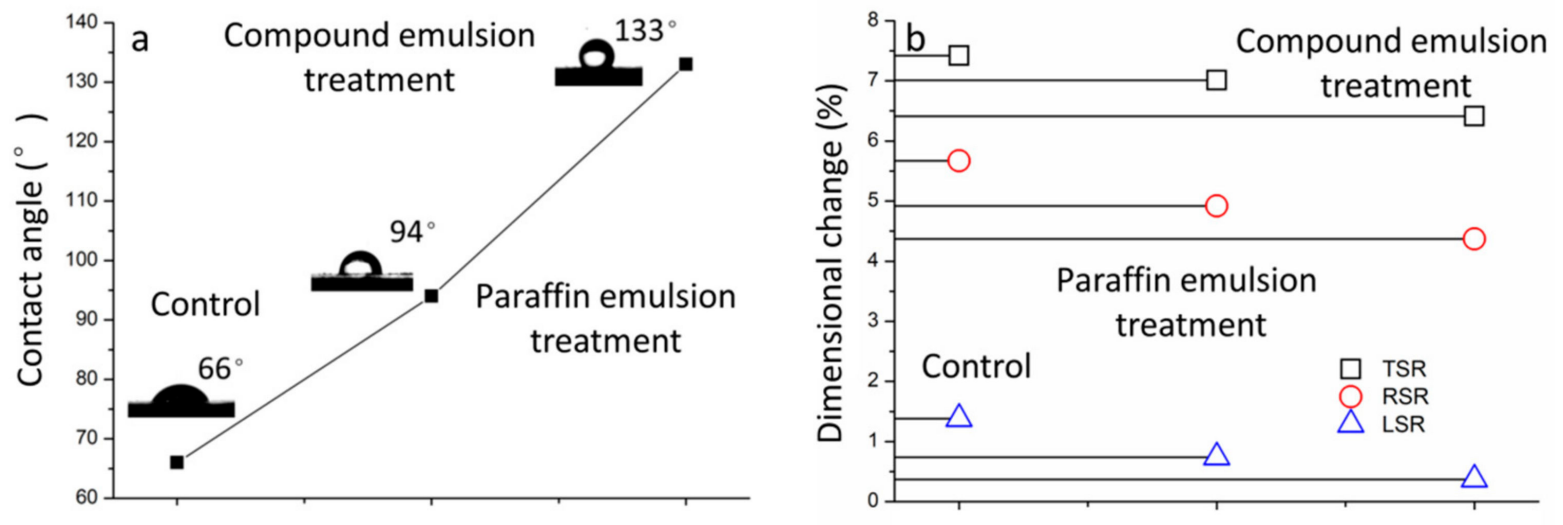
(**a**) Water contact angle and (**b**) dimensional changes of untreated (control) and treated wood.

To further clarify the influence of compound emulsion on the hydrophobicity of wood, the water contact angle (WCA), dimensional stability and microstructure of treated wood were investigated. According to the optimal conditions, the solid content of compound emulsion was 13 wt %, which was determined as the concentration of paraffin emulsion in this part to avoid the impact of solid content on the test. WCA is indicative of the hydrophobicity of wood [29]. As shown in Figure 5a, WCAs in all emulsion treated wood samples increased obviously compared to the control sample (untreated wood). After paraffin emulsion impregnation, the WCA increased to 94°, indicating a considerable raise in hydrophobicity due to the paraffin penetration. Moreover, the WCA in compound emulsion treated wood was 133°. This suggested that the hydrophobicity could be further improved by the addition of acrylate emulsion. This is because the acrylate emulsion could form a hydrophobic film in the wood via a capillary force during the post-treatment process (drying for demulsification). With the help of hydrophobic paraffin, this microstructure or distribution could protect the wood against water penetration and decrease surface wettability.

Generally, the dimensional change can decrease after the hydrophobic treatment of wood. This is mainly due to the penetration of hydrophobic materials in pores, which can prevent water absorption and dimensional swelling. As expected, paraffin emulsion showed a positive effect on dimensional stability parameters, including TSR, RSR and LSR (Figure 5b). This is in agreement with the result from WCA tests, indicating the positive effect of acrylate emulsion on dimensional stability.

### 3.6. Microstructure

**Figure 6:**
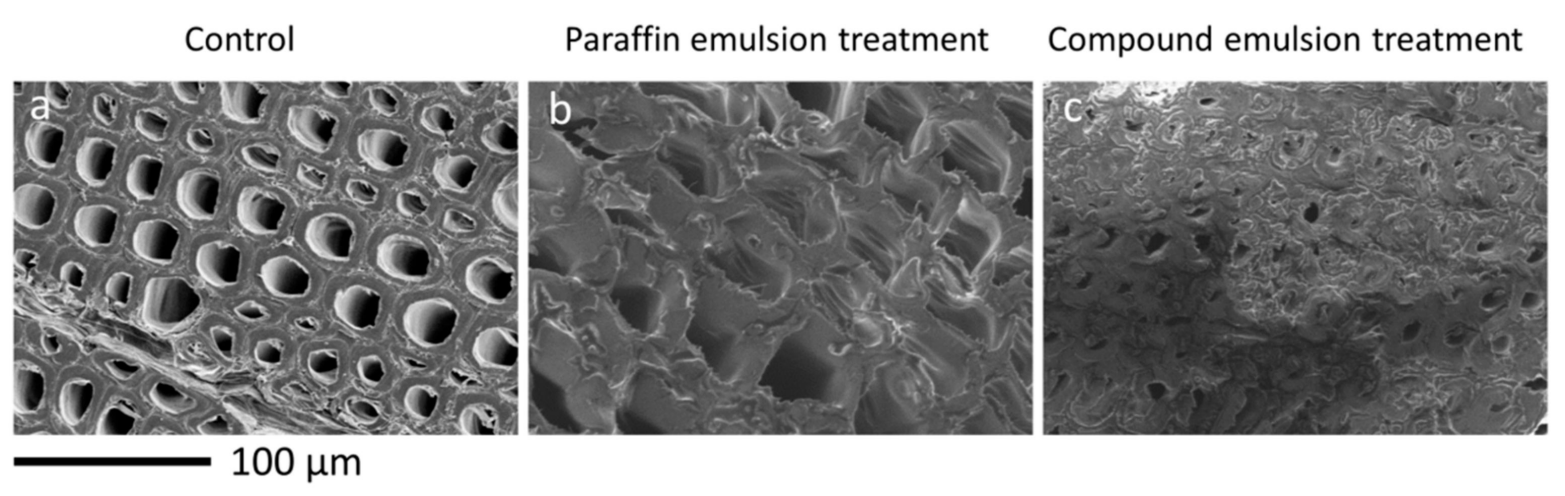
SEM micrographs of untreated (control) and treated wood samples: (**a**) control (**b**) paraffin emulsion treatment and (**c**) compound emulsion treatment.

Microstructural observations could provide a better understanding of the wood treatment process. SEM micrographs of untreated and emulsion treated wood specimens show perceptible differences (Figure 6). Some lumens are partially or totally filled with paraffin or acrylate. Compared to the control sample (Figure 6a), the paraffin emulsion treated wood displayed smooth cell walls owing to the bulking effect of liquid paraffin penetration and coverage (Figure 6b). This could cause water rejection in cell walls [5,30]. With the incorporation of acrylate emulsion, the filling effect in treated wood appeared to be more remarkable (Figure 6c). The polymer colloidal particles could effectively fill the pores in wood and form hydrophobic films via capillary forces during the drying process. As a result, the synergistic effect (bulking effect and filling effect) of paraffin and acrylate plays an important role in improving the hydrophobicity and dimensional stability of compound emulsion treated wood.

## 4. Conclusions

Response surface methodology was successfully used to investigate the relationship between hydrophobicity and wood treatment parameters. The paraffin emulsion concentration and acrylate emulsion percentage had significant influences on water absorption (WA) and mass percentage gain (MG). The WA decreased obviously with increasing acrylate emulsion percentage. SEM observations confirmed that this phenomenon was caused by a better filling effect of the compound emulsion via the formation of a completely hydrophobic film in the wood structure. The correlation models for WA and MG showed a good prediction due to the near linear distribution in the normal probability plot of residuals. The optimal conditions (5.57% paraffin emulsion concentration, 20% acrylate emulsion percentage, and 10 min treatment time) provided by RSM were acceptable for predicting MG and WA. Compared to untreated (66°) and paraffin emulsion treated wood (94°), the wood treated with compound emulsion under optimal conditions showed the highest WCA (133°) and better dimensional stability. This could be ascribed to a synergistic effect of paraffin and acrylate (bulking effect and filling effect), and the consequent formation of hydrophobic films in wood.

## Figures and Tables

**Table 1:** Different combinations and droplet sizes in compound emulsions.

Compound Emulsions	Paraffin Emulsion Concentration (%)	Acrylate Emulsion Percentage (%)	Droplet Size (nm)
CE-2-10	2	10	169.3
CE2-20	2	20	164.3
CE5-10	5	10	184.6
CE5-20	5	20	176.5
CE8-10	8	10	190.2
CE8-20	8	20	184.0

**Table 2:** Parameters and levels used for compound emulsion treated wood.

Parameter Codes			Levels	
		−1	0	1
Paraffin emulsion concentration (%)	A	2	5	8
Acrylate emulsion percentage (%)	B	0	10	20
Treatment time (min)	C	10	25	40

**Table 3:** Experimental conditions and results for compound emulsion treated wood.

Run Order	Factors			MG (%)	WA (%)
	Paraffin Emulsion Concentration (%)	Acrylate Emulsion Percentage (%)	Treatment Time (min)		
1	5.00	0.00	40.00	8.90	44.10
2	8.00	20.00	25.00	16.82	35.42
3	5.00	10.00	25.00	15.64	40.45
4	2.00	10.00	40.00	9.53	47.61
5	5.00	10.00	25.00	11.09	40.98
6	8.00	10.00	40.00	18.53	39.73
7	5.00	10.00	25.00	14.68	43.17
8	8.00	0.00	25.00	14.46	40.88
9	2.00	20.00	25.00	12.86	44.30
10	5.00	0.00	10.00	8.80	46.54
11	2.00	0.00	25.00	4.80	52.60
12	5.00	20.00	40.00	14.51	34.98
13	5.00	20.00	10.00	17.07	37.10
14	5.00	10.00	25.00	15.34	42.37
15	5.00	10.00	25.00	12.52	42.72
16	8.00	10.00	10.00	18.96	39.84
17	2.00	10.00	10.00	8.46	46.13

**Table 4:** Analysis of variance for the MG model.

Source	Sum of Squares	Degrees of Freedom	Mean Square	*F*-Value	*P*-Value Probability > *F*
Model	221.80	6	36.97	11.15	0.0006 ^a^
A—Paraffin emulsion concentration	137.12	1	137.12	41.36	<0.0001 ^a^
B—Acrylate emulsion percentage	73.81	1	73.81	22.26	0.0008 ^a^
C—Treatment time	0.41	1	0.41	0.12	0.7311 ^n^
AB	8.12	1	8.12	2.45	0.1486 ^n^
AC	0.56	1	0.56	0.17	0.6891 ^n^
BC	1.77	1	1.77	0.53	0.4819 ^n^
Residual	33.16	10	3.32		
Lack-of-fit	17.66	6	2.94	0.76	0.6372 ^n^

Note: ^n^ Insignificant (*P* > 0.1); ^a^ Highly significant (*P*< 0.01); ^b^ Moderately significant (0.01 < *P* < 0.05); ^c^ Significant (0.05 < *P* < 0.1).

**Table 5:** ANOVA parameters for the MG model.

Response	Standard Deviation	R^2^	Adjusted R^2^
MG	1.83	0.8290	0.7895

**Table 6:** Analysis of variance for the WA model.

Source	Sum of Squares	Degrees of Freedom	Mean Square	F-Value	P-value Probability > F
Model	285.64	6	47.61	12.77	0.0004 ^a^
A—Paraffin emulsion concentration	151.12	1	151.12	40.55	<0.0001 ^a^
B—Acrylate emulsion percentage	130.57	1	130.57	35.04	0.0001 ^a^
C—Treatment time	1.27	1	1.27	0.34	0.5720 ^n^
AB	2.02	1	2.02	0.54	0.4789 ^n^
AC	0.63	1	0.63	0.17	0.6892 ^n^
BC	0.026	1	0.026	6.870 × 10^−3^	0.9356 ^n^
Residual	37.27	10	3.73		
Lack-of-fit	31.82	6	5.30	3.89	0.1046 ^n^

Note: ^n^ Insignificant (*P* > 0.1); ^a^ Highly significant (*P* < 0.01); ^b^ Moderately significant (0.01 < *P* < 0.05); ^c^ Significant (0.05 < *P* < 0.1).

**Table 7:** ANOVA parameters for the WA model.

Response	Standard Deviation	R^2^	Adjusted R^2^
WA	1.75	0.8763	0.8478

**Table 8:** Predicted and experimental values obtained under optimum conditions.

Index	MG (%)	WA (%)
Predicted value	17.6	37.9
Experimental value	19.5	42.7
Error	9.7	11.2
